# Short-term improvements in diet quality in people newly diagnosed with type 2 diabetes are associated with smoking status, physical activity and body mass index: the 3D case series study

**DOI:** 10.1038/s41387-020-0128-3

**Published:** 2020-07-13

**Authors:** Emily Burch, Lauren T. Williams, Lukman Thalib, Lauren Ball

**Affiliations:** 1 Menzies Health Institute Queensland, Griffith University, Gold Coast, 4215 Australia; 2 Department of Public Health, College of Health Sciences, QU Health, Qatar University, Doha, Qatar

**Keywords:** Nutrition, Type 2 diabetes

## Abstract

**Background:**

Dietary intake impacts glycaemic control through its effect on weight and glucose-insulin homeostasis. Early glycaemic control is associated with improved outcomes and reduced mortality for people with type 2 diabetes (T2D). To date, the diet quality of people with T2D has only been studied cross-sectionally. The objective of this paper is to quantify short-term improvements in diet quality and to identify factors associated with improvements after T2D diagnosis among participants in the 3D study.

**Methods:**

This paper presents data from the 3D study of 225 Australian adults, newly diagnosed with T2D. Telephone interviews collected demographic, diet, physical and health data at baseline and 3 months. Diet quality was assessed using the Dietary Approaches to Stop Hypertension (DASH) tool to examine short-term changes in diet quality after diagnosis. Participants were categorised into two groups: those who improved their diet quality by 3 months (increase in DASH score of 3 or more) and those who did not. Factors associated with change in DASH scores were clinically and statistically evaluated.

**Results:**

The 3D cohort was comparable to Australian cohorts with diabetes by gender and body mass index (BMI) but differed by age, remoteness and socioeconomic status. Mean (SD) baseline DASH score was 24.4 (4.7), in the midrange of possible scores between 8 and 40. One third of participants improved their DASH score by 3-months. This group had lower diet quality (*p* < 0.001), lower BMI (*p* = 0.045), higher physical activity levels (*p* = 0.028) and were less likely to smoke (*p* = 0.018) at baseline.

**Conclusions:**

Diet quality changes after diagnosis do not appear to be associated with demographic characteristics but were associated with lifestyle behaviours. Strategies targeted at better supporting smokers, those with low physical activity and higher BMI are required. Future research should investigate how the diet quality changes people make around time of diagnosis are related to long-term health outcomes.

## Introduction

Lifestyle modification is the first line of treatment for people newly diagnosed with type 2 diabetes (T2D)^1^. Dietary intake impacts glycaemic control through its effect on weight and glucose-insulin homeostasis^2^. Early, tight glycaemic control is associated with improved outcomes and reduced mortality for people with T2D, regardless of how the disease is managed in the future^3^. It is therefore important to understand the changes that people with T2D make to improve diet quality immediately after diagnosis.

Intervention studies have shown several dietary patterns to be effective in improving glycaemic control and other health outcomes in individuals with T2D such as hypertension and lipid profiles^4–6^. These include; the Mediterranean diet, the Dietary Approaches to Stop Hypertension (DASH) diet, low-glycaemic index diet, moderately low-carbohydrate diet, vegetarian and vegan diets, and general healthy eating diets^1,4^. Dietary management guidelines for T2D suggest that personal preferences, cultural backgrounds, and metabolic goals should be considered when recommending one dietary pattern over another as no particular pattern will suit all people with T2D^7^. The DASH dietary pattern has been shown to be beneficial for glycaemic control, blood pressure, body weight, waist circumference and lipid levels in people with T2DM and was therefore the dietary pattern used to assess diet quality in this study^8,9^.

Despite the well-recognised impact diet quality can have on diabetes-related health outcomes, our systematic review has shown that, people with T2D have low-quality diets because they do not meet fruit, vegetable, whole-grain or low-fat dairy food group recommendations and meet or exceed the red meat and meat alternatives food group recommendations^10^. This suggests strategies to support healthy eating for people with T2D are still required. However, this review was limited by the fact that the included studies were all of cross-sectional design^10^. Prospective designs are required to investigate what dietary changes people make immediately after T2D diagnosis and the demographic and health characteristics related to these changes^11^. Interventions can then be designed to support these people to improve diet quality to achieve early, tight glycaemic control.

The objective of this paper is to quantify short-term improvements in diet quality and to identify factors associated with improvements after T2D diagnosis among participants in the 3D study. The 3D study: “How does diet change with a diagnosis of diabetes” used a case-series design to follow a national sample of Australians newly diagnosed with T2D over a 12-month period.

## Methods

### Study design and participants

Methodological details of the 3D study have been published elsewhere^11^. Briefly, adults over the age of 18 years diagnosed with T2D in the 6-months prior to recruitment were monitored through five interviewer-administered telephone surveys at baseline, 3, 6, 9 and 12 months. Informed consent was obtained from all participants. Dietary, physical and other health characteristics were collected at each time point. Demographic characteristics were collected at baseline only. This paper presents data collected at baseline and 3 months only. The 3D study is registered with the Australian New Zealand Clinical Trials Registry (ANZCTR) (ref: ACTRN12618000375257) and was approved by the Griffith University Human Research Ethics Committee (ref: 2017/951).

### Data collection

Data were collected using interviewer-administered telephone surveys. Surveys were ~30 min in duration and were conducted by Accredited Practicing Dietitians (APD). Dietary intake data were entered by the APD into the Australian version of the Automated Self-Administered 24-h Dietary Assessment Tool (ASA-24). All other data were recorded by the interviewer in an online survey management system: www.limesurvey.org.

### Demographic characteristics

Demographic characteristics collected at baseline included age, gender, highest education level, living arrangement, self-selected social class, language, indigenous status and household income. Response options were consistent with categories used in the national census by the Australian Bureau of Statistics (ABS). Participants were assigned an Accessibility/Remoteness Index of Australia (ARIA) score which divides Australia into five classes of remoteness on the basis of a measure of relative access to services^12^. Participants were categorised into Socio-Economic Indexes for Area (SEIFA) quintiles based on their postcode, which indicates the socio-economic advantage/disadvantage of areas using information from the ABS census^13^. Demographic characteristics of the 3D sample were compared with the Australian Living with Diabetes (LWD) study^14^, the 2015 Management and Impact for Long-term Empowerment and Success (MILES-2) study^15^ and the national 2014–15 Australian Health Survey to examine representativeness of the sample^16^.

### Health characteristics

Health characteristics collected at every time point included smoking status, weight and waist circumference (participants were mailed a tape measure for this purpose). Height was collected at baseline only. The World Health Organization (WHO) classification system for body mass index (BMI) and gender specific waist circumference were used to categorise into groups^17^. Physical activity levels were measured through the International Physical Activity Questionnaire (IPAQ) short form^18^. Information on medication use (name of medication and dosage) including over the counter and complementary medicines were collected. Mental health was assessed using the internationally validated Kessler Psychological Distress Scale (K10) questionnaire^19^. Diabetes characteristics collected at baseline included date of T2D diagnosis and one question related to whether the participant had been told they had prediabetes before they received a formal T2D diagnosis from their General Practitioner. Health and diabetes characteristics of the 3D sample were compared to Australian studies: the LWD^14^ and the 2015 MILES-2^15^.

### Diet quality (DASH)

24-hour dietary recall data acquired through the ASA-24 was automatically entered into the dietary analysis programme FoodWorks^20^. FoodWorks data were then used to calculate DASH scores between 8 and 40 points (40 points representing optimal accordance with the DASH dietary pattern) using the standard scoring tool^21^. DASH score was calculated by summing the number of daily servings of seven dietary components; fruits, vegetables, nuts and legumes, whole-grains, low-fat dairy, red and processed meats, sodium and sugar-sweetened beverages^21^.

### Change in diet quality

Change in diet quality was calculated by subtracting participants DASH scores at baseline from the score at 3 months. Participants were categorised into two groups; those who improved their DASH score by at least 3 points (‘improvers’) and those who improved by 2 or fewer points, maintained their DASH score or decreased their DASH score (‘maintainers’). An increase of 3 points was deemed as the smallest clinically significant change based on findings from a 20-year longitudinal study of over 40,000 adults that showed an increase of at least 3 points to significantly influence long-term glycaemic control as assessed by glycated haemoglobin (HbA1c)^22^.

### Data analysis

Data cleaning was conducted to ensure accuracy and plausibility. Ten percent of the records were double checked for accuracy of data entry at the conclusion of each data collection point. Data were then cleaned and checked for errors by a data co-ordinator within the research team. The range of values for each variable were then assessed for plausibility and outliers were double checked for their accuracy. Sample representativeness was explored by comparing available demographic and health characteristics of the sample with national data^14–16^ using Pearson’s *χ*^2^ goodness of fit tests. For longitudinal analyses, crude associations between the baseline demographic, health and diet related behavioural characteristics were investigated for all participants. Pearson’s *χ*^2^ and Fisher’s Exact tests were used to compare categorical variables. Independent sample *t*-tests and One-way ANOVAs were used to compare normally distributed continuous variables and Mann–Whitney *U* and Kruskal–Wallis tests were used to compare non-normally distributed continuous variables.

## Results

### Study sample

**Fig. 1:**
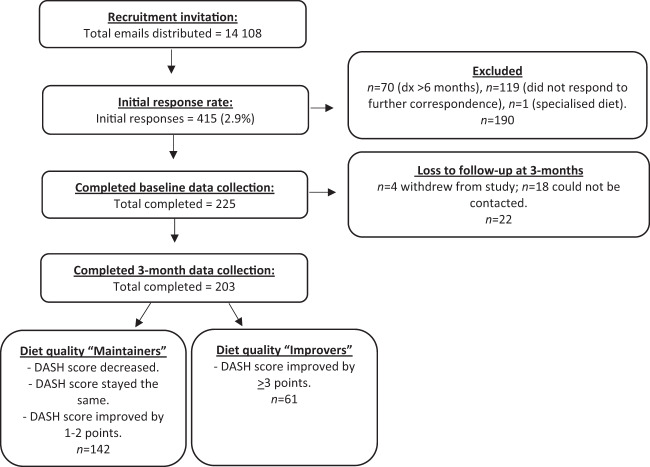
Flow chart outlining 3D study participant recruitment, exclusions, loss to follow-up and diet quality group allocation in the first 3-months of the study. Recruitment, exclusions, loss to follow-up and participant allocation during the first 3-months of the 3D study.

A flow chart outlining recruitment and retention and participant distribution between ‘improvers’ and ‘maintainers’ between baseline and 3 months of the 3D study is outlined in Fig. 1. Between May 2018 and August 2018, a total of 14,108 individuals who had been diagnosed with T2D in the previous 6 months were sent an email from Diabetes Australia inviting them to participate in the study. Four hundred and fifteen individuals contacted the researchers to express interest in the study (2.9% response rate), 190 of whom were ineligible. Reasons for ineligibility included; a diagnosis of diabetes above 6 months prior to recruitment (*n* = 70), non-responsive to request for more details (*n* = 119) or on a restricted, specialised diet due to a medical condition (*n* = 1). Two hundred and twenty-five participants completed all components of the baseline survey, 203 of whom also completed the 3-month survey. Four participants withdrew from the study at 3 months and 18 were uncontactable after two emails, two text messages and one phone call request to schedule an interview time (90.2% retention rate). Participants who completed baseline but not the 3-month data collection had a lower mean age (*p* = 0.006), less time since diagnosis (*p* = 0.018) and a higher baseline DASH score (*p* = 0.029; data not shown).

Supplementary Table 1 describes the demographic characteristics of the 3D sample at baseline. There were more males than females in the 3D sample, consistent with the three comparison cohorts. 3D participants were younger than the national cohort (*p* < 0.001), which is to be expected given the recency of their diagnosis. Consistent with the LWD^14^ and MILES-2 cohorts^15^, proportions of Aboriginal Torres Strait Islander people were low, with only two people identifying as having this background. The remoteness of participants differed between the 3D sample and the national cohort (*p* < 0.001), with a lower proportion of 3D participants in inner-regional (13.8% vs 21.6%), and outer-regional areas (7.6% vs 13.4%) than the national cohort^16^. The socioeconomic status of participants differed between the 3D sample and the national cohort (*p* < 0.001), with fewer 3D participants in the lowest socio-economic group compared with the national cohort (20.2% vs 30.6%). The majority of 3D participants identified as either upper or middle class (79.0%). Nearly two thirds of participants had a gross yearly income of at least $50 000 (63.9%).

A national map that outlines the proportion of participants in the 3D sample from each Australian state compared to 2019 Diabetes Australia data of people with T2D is provided in Supplementary Fig. 1^23^. The 3D participant distribution differed from the Diabetes Australia database of all people with diabetes (*p* < 0.001). Upon visual inspection, the 3D study had a higher proportion of participants from Queensland (33.0% vs 19.1%), and lower from New South Wales (19.6% vs 32.3%) than the Diabetes Australia data.

Supplementary Table 2 describes the health characteristics of the 3D sample at baseline. The mean time from diagnosis to baseline data collection was 114.5 ± 41.1 days (~3.8 months). The majority of 3D participants had a BMI in the obese range (59.4%) which was consistent with the MILES-2 cohort (*p* = 0.179), but not the LWD (*p* = 0.017). More than 90% of participants had a waist circumference higher than the national sex-specific recommendations^17^. The highest proportion of 3D participants had a low K10 score (51.6%), a moderate physical activity level (54.2%) and had never smoked (61.3%).

### Diet quality

Table 1 shows the diet quality of the 3D sample at baseline. The mean (SD) DASH score of the group was 24.4 ± 4.7 with no significant difference between males and females (*p* = 0.623). Mean energy (kJ) intake per day at baseline was significantly higher for males than females (*p* < 0.001). Males had a higher baseline vegetable serve intake compared with females (*p* = 0.004), and a higher whole-grain intake (*p* = 0.034). Table 2 describes DASH score changes between baseline and 3 months of the 3D study. Slightly less than a third (31%) of participants improved their DASH score to the clinically significant outcome of 3 points or greater.

**Table 1:** Dietary intake characteristics at baseline of 3D study participants.

Diet characteristic	Males *n* = 126 (56.0%)	Females *n* = 99 (44.0%)	All *n* = 225 (100.0%)	*P* value
Mean ± SD DASH score (range)	24.3 ± 4.6 (12.0–36.0)	24.6 ± 4.7 (14.0–37.0)	24.4 ± 4.7 (12.0–37.0)	0.623
Food group serves/d (range)^a^				
Fruit	0.8 (0.0–5.7)	0.7 (0.0–3.7)	0.8 (0.0–5.7)	0.601
Vegetables	4.1 (0.0–11.6)	3.2 (0.0–13.3)	3.7 (0.0–13.3)	0.004
Nuts and legumes	0.6 (0.0–5.9)	0.5 (0.0–4.3)	0.5 (0.0–5.9)	0.831
Whole-grains	2.4 (0.0–10.0)	1.7 (0.0–6.2)	2.1 (0.0–10.0)	0.034
Low-fat dairy	0.4 (0.0–3.9)	0.4 (0.0–4.0)	0.4 (0.0–4.0)	0.558
Sodium (mg/d)	2377.3 (298.7–8854.2)	2069.9 (545.5–7435.7)	2242.0 (298.7–8854.2)	0.070
Red/processed meats	1.2 (0.0–7.1)	1.1 (0.0–5.7)	1.1 (0.0–7.1)	0.882
SSBs	0.1 (0.0–1.8)	0.0 (0.0–1.6)	0.1 (0.0–1.8)	0.084
% recommendations met^b^				
Fruit serves/d	–	–	17.8	–
Vegetable serves/d	–	–	31.6	–
Mean ± SD energy intake (KJ/d)	7809.1 ± 2796.5	6233.2 ± 1871.5	7115.7 ± 2551.7	<0.001
Energy intake range (KJ/d)	1803.9–16285.3	2685.1–11354.8	1803.9–16285.3	–

Statistics: Kruskal–Wallis test.

*SSBs* sugar-sweetened beverages, *KJ/d* kilojoules per day, *serves/d* serves per day, *DASH* Dietary Approaches to Stop Hypertension, *mg/d* milligrams per day.

^a^Data shown in lowest – highest range.

^b^Based on Australian Guide to Healthy Eating recommendations.

**Table 2:** Diet quality change between baseline and 3 months for *n* = 203 3D participants.

DASH change group	DASH score change at 3 months	*n* (%)	Mean baseline DASH score ± SD (range)
‘Improvers’	Improved by >5 points	29 (14.3)	19.7 ± 4.7 (12–32)
	Improved by 5 points	16 (7.9)	21.7 ± 4.1 (13–30)
	Improved by 4 points	3 (1.5)	17.7 ± 3.5 (14–21)
	Improved by 3 points	15 (7.4)	23.9 ± 4.3 (15–33)
Total		63 (31.0)	

‘Maintainers’	Improved by 2 points	17 (8.4)	23.4 ± 4.6 (17–32)
	Improved by 1 point	9 (4.4)	25.1 ± 2.3 (22–29)
	No change	18 (8.9)	24.3 ± 4.6 (17–34)
	Decreased by 1 point	15 (7.4)	23.0 ± 2.8 (16–28)
	Decreased by 2 points	16 (8.4)	25.4 ± 3.2 (19–32)
	Decreased by 3 points	10 (4.9)	24.0 ± 3.6 (20–30)
	Decreased by 4 points	14 (6.9)	26.0 ± 3.8 (19–32)
	Decreased by 5 points	10 (4.9)	27.1 ± 4.3 (23–37)
	Decreased by > 5 points	31 (15.3)	28.2 ± 3.3 (21–37)
Total		140 (69.0)	

Baseline diet quality according to DASH score change (improvers vs maintainers) is outlined in Table 3. Participants who improved their diet quality had a lower mean baseline DASH score (*p* < 0.001). The lower mean baseline DASH score stemmed from a lower intake of fruit (*p* < 0.001), vegetable (*p* = 0.026), low-fat dairy (*p* = 0.003) and wholegrain serves (*p* = 0.016), and higher mean intake of sugar-sweetened beverages serves (*p* < 0.001), red and processed meats (*p* = 0.033) and nuts and legumes serves (*p* < 0.037) when compared with participants who did not improve their diet quality.

**Table 3:** Baseline diet characteristics according to DASH score change by 3 months.

DASH change group			
Diet characteristic	‘Improvers’ *n* = 61 (30.1%)	‘Maintainers’ *n* = 142 (69.9%)	*P* value
Mean ± SD DASH score (range)			
Males	21.4 ± 5.2 (12.0–33.0)	25.1 ± 4.0 (16.0–37.0)	<0.001
Females	20.7 ± 3.9 (14.0–28.0)	25.8 ± 4.1 (17.0–37.0)	<0.001
Males and females	21.1 ± 4.7 (12.0–33.0)	25.4 ± 4.0 (16.0–37.0)	<0.001
Mean ± SD (serves/d) food groups (range)			
Fruit	0.4 ± 0.7 (0–4.1)	0.9 ± 1.0 (0.0–4.1)	<0.001
Vegetables	3.1 ± 2.4 (0.0–11.6)	3.9 ± 2.6 (0.0–13.3)	0.033
Nuts and legumes	0.7 ± 1.7 (0.0–10.4)	0.5 ± 0.8 (0.0–4.25)	0.037
Whole-grains	1.7 ± 1.8 (0.0–7.7)	2.3 ± 2.0 (0.0–9.5)	0.014
Low-fat dairy	0.2 ± 0.5 (0.0–2.8)	0.5 ± 0.8 (0.0–4.0)	0.004
Sodium	2317.8 ± 1173.9 (545.5–5305.8)	2279.1 ± 1323.6 (298.7–8854.2)	0.638
Red/processed meats	1.4 ± 1.5 (0.0–7.1)	1.0 ± 1.4 (0.0–6.4)	0.053
SSBs	0.2 ± 0.4 (0.0–1.8)	0.0 ± 0.2 (0.0–1.2)	<0.001

Statistics: Kruskal–Wallis test for continuous variables.

*DASH* dietary approaches to stop hypertension, *serves/d* serves per day, *SSBs* sugar sweetened beverages.

There were no significant differences in baseline demographic characteristics (including sex, age, education level, living situation, income, social class, remoteness and socio-economic group) between participants who improved their diet quality and those who did not, as shown in Table 4. Table 5 outlines the differences in baseline health characteristics between the two groups. There was a significant difference between the two groups for physical activity levels (*p* = 0.028) and smoking status (*p* = 0.018). Participants who made early improvements to diet quality reported higher levels of physical activity at baseline (19.7% vs 7.1%), were less likely to be ‘current’ smokers (6.6% vs 12.0%) and more likely to be ex-smokers (45.9% vs 26.1%) compared with those that did improve their diet quality. While there was no difference in BMI category, there was an overall difference in mean BMI (*p* = 0.045). A further gender stratified analysis showed that the significant effect was only for the male participants (*p* = 0.048). No other differences in health characteristics were found between the groups.

**Table 4:** Demographic characteristics according to DASH score change by 3 months.

DASH change group				
Demographic characteristic	‘Improvers’ *n* = 61 (30.1%)	‘Maintainers’ *n* = 142 (69.9%)	Total *n* = 203 (100.0%)	*P* value
Sex *n* (%)				
Male	36 (59.0)	77 (54.2)	126 (56.0)	0.529
Female	25 (41.0)	65 (45.8)	99 (44.0)	
Age *n* (%)				
<55 years	20 (32.8)	45 (31.7)	65 (32.0)	0.752
55–65 years	20 (32.8)	54 (38.0)	74 (36.5)	
>65 years	21 (34.4)	43 (30.3)	64 (31.5)	
Mean ± SD age (years)	59.0 ± 10.3	59.0 ± 10.3	–	0.628
Highest education *n* (%)				
Higher education degree	25 (41.7)	45 (32.1)	70 (35.0)	0.328
Diploma/certificate	18 (30.0)	56 (40.0)	74 (37.0)	
No post-school education	17 (28.3)	39 (27.9)	56 (28.0)	
Living situation *n* (%)				
Partner/spouse	41 (68.3)	95 (67.9)	136 (68.0)	0.995
Other	7 (11.7)	17 (12.1)	24 (12.0)	
Nobody	12 (20.0)	28 (20.0)	40 (20.0)	
Gross yearly income *n* (%)				
<$50,000	17 (34.7)	44 (35.5)	61 (35.3)	0.801
$50,001–100,000	17 (34.7)	37 (29.8)	54 (31.2)	
>$100,001	15 (30.6)	43 (34.7)	58 (33.5)	
Social class *n* (%)				
Upper/Middle	42 (84.0)	83 (76.9)	125 (79.1)	0.304
Working	8 (16.0)	25 (23.1)	33 (20.9)	
Remoteness *n* (%)				
Major city	45 (73.8)	106 (74.6)	151 (74.4)	0.955
Inner regional	10 (16.4)	21 (14.8)	31 (15.3)	
Outer regional	5 (8.2)	11 (7.8)	16 (7.8)	
Remote/very remote	1 (1.6)	4 (2.8)	5 (2.5)	
Socioeconomic group *n* (%)				
1 (lowest SES)	16 (26.2)	24 (17.0)	40 (19.7)	0.544
2	14 (23.0)	31 (22.0)	45 (22.2)	
3	8 (13.1)	28 (19.8)	36 (17.7)	
4	12 (19.7)	29 (20.6)	41 (20.2)	
5	11 (18.0)	29 (20.6)	41 (20.2)	

Statistics: *χ*^2^ test or Fisher’s Exact test for categorical and Kruskal–Wallis test for continuous variables.

*DASH* Dietary Approaches to Stop Hypertension, *SES* socio-economic status.

**Table 5:** Baseline health characteristics according to DASH score change by 3 months.

DASH change group				
Health characteristic	‘Improvers’ *n* = 61 (30.1%)	‘Maintainers’ *n* = 142 (69.9%)	Total *n* = 203 (100.0%)	*P* value
Smoking status *n* (%)				
Smoker	4 (6.6)	17 (12.0)	21 (10.3)	
Ex-smoker	28 (45.9)	37 (26.1)	65 (32.0)	
Never smoked	29 (47.5)	88 (62.0)	117 (57.7)	0.018
Diagnosed with pre-diabetes *n* (%)				
Yes	29 (49.2)	61 (43.9)	90 (45.4)	
No	30 (50.8)	78 (56.1)	108 (54.6)	0.496
Using diabetic medication *n* (%)				
Yes	45 (73.8)	86 (60.6)	131 (64.5)	0.071
No	16 (26.2)	56 (39.4)	72 (35.5)	
IPAQ *n* (%)				
Low	18 (29.5)	52 (36.6)	70 (34.5)	
Moderate	31 (50.8)	80 (56.3)	111 (54.7)	
High	12 (19.7)	10 (7.1)	22 (10.8)	0.028
K10 score *n* (%)				
Low	35 (57.4)	67 (47.9)	102 (50.7)	
Moderate	15 (24.6)	38 (27.1)	53 (26.5)	
High	5 (8.2)	20 (14.3)	25 (12.4)	
Very high	6 (9.8)	15 (10.7)	21 (10.4)	0.540
BMI class *n* (%)				
Healthy	11 (18.0)	17 (12.1)	28 (13.9)	
Overweight	19 (31.2)	37 (26.2)	56 (27.7)	
Obese	31 (50.8)	87 (61.7)	118 (58.4)	0.312
Mean ± SD BMI (kg/m^2^)				
Males	29.5 ± 5.3	32.0 ± 6.5		0.048
Females	32.1 ± 6.7	33.1 ± 6.9	–	0.481
Males and females	30.5 ± 6.0	32.5 ± 6.7		0.045
Mean ± SD waist circumference (cm)				
Male	106.8 ± 12.5	111.4 ± 15.9		0.207
Female	105.8 ± 17.4	108.3 ± 17.9	–	0.504
Males and females	106.4 ± 14.6	110.0 ± 16.9		0.160
Waist circumference *n* (%)				
Meets recommendations	7 (13.0)	11 (8.3)	–	
Exceeds recommendations	47 (87.0)	121 (91.7)		0.332
Mean ± SD weight (kg)				
Males	91.1 ± 18.0	99.1 ± 21.7		0.077
Females	84.1 ± 20.8	88.4 ± 19.5	–	0.273
Males and females	88.2 ± 19.3	94.3 ± 21.3		0.070
Mean ± SD height (m)				
Males	1.8 ± 0.1	1.8 ± 0.1		0.965
Females	1.6 ± 0.1	1.6 ± 0.1	–	0.127
Males and females	1.7 ± 0.1	1.7 ± 0.1		0.730
Mean ± SD energy intake (KJ/d) (range)				
Males	8374.7 ± 3251.8 (1886.7–16285.3)	7664.0 ± 2640.0 (1803.9–13289.7)	–	0.358
Females	6082.7 ± 1909.3 (3335.5–11354.8)	6303.8 ± 1809.6 (2929.1–10536.4)		0.457
Males and females	7435.4 ± 2986.4 (1886.7–16285.3)	7041.4 ± 2388.7 (1803.9–13289.7)		0.600

Statistics: *χ*^2^ test or Fisher’s exact test for categorical and Kruskal–Wallis test for continuous variables.

*DASH* Dietary Approaches to Stop Hypertension, *IPAQ* International Physical Activity Questionnaire, *BMI* body mass index, *cm* centimeters, *m* metre, *kg* kilogram, *KJ* kilojoule, *d* day, *serves/d* serves per day, *SSBs* sugar sweetened beverages.

## Discussion

To our knowledge, this is the first study to examine how diet quality changes over time immediately following T2D diagnosis. Given the evidence that early tight glycaemic control is associated with better health outcomes, the findings of the 3D case series study provides an understanding of the nature of dietary change and the factors associated with these changes. Demographic characteristics were not associated with diet quality changes after diagnosis. Those who made diet quality improvements in our study period tended to have lower diet quality, did not currently smoke, had a lower BMI and higher physical activity levels at baseline.

Diet quality is an important component of T2D management, particularly when newly diagnosed as outlined in practice guidelines^1,3,4^. Dietary modification strategies aimed at improving body weight and/or diet quality can contribute to improvements in glycaemic control, blood pressure, weight and lipid levels in people with T2D^24^. Meaningful diet quality change only occurred in about one third of the study sample during the observation period of this study. It is possible that individuals who did not make meaningful diet quality change during this period previously made changes when diagnosed with pre-diabetes. However, another analysis of the 3D study sample showed there were no differences in diet quality at baseline between those diagnosed with pre-diabetes and those who had not received a pre-diabetes diagnosis^25^. Another possibility is that some participants made changes immediately after receiving a diagnosis but before completing the baseline survey of the 3D study. The fact that those who did not improve started the study with a higher DASH score supports this supposition.

Diet quality has been shown to be inversely associated with rates of obesity^26–29^. In the present study, men who did not improve their diet quality by 3 months had a significantly higher baseline BMI than men who improved their diet quality (*p* = 0.048). This finding is consistent with cross-sectional data from 2018 that found an inverse association between diet quality (measured by DASH score) and obesity in 211 Chinese adults (54.5% male) with T2D^27^. Similarly, a 2013 cross-sectional study of 99 adults (27.3% male) with T2D in the United States (US) found that individuals who were of normal weight or overweight, had a significantly higher diet quality (measured by Healthy Eating Index score) than those who were obese^29^. The present study has advanced upon these cross-sectional studies by investigating changes in diet quality over time, suggesting that male patients who have a higher BMI at diagnosis may find dietary change more difficult than those who start off with lower mean BMI. Those men with higher BMI at diagnosis may require additional support to improve their diet quality after being diagnosed with T2D. It is interesting that this relationship was not observed with women, that is, their change in diet quality was independent of baseline BMI.

Physical activity levels are a critical focus for glycaemic management and overall health of people with T2D^30^. The present study found that participants who improved their diet quality by 3 months had higher overall activity levels at baseline than those who did not improve their diet quality (*p* = 0.028). A 2012 cross-sectional study of 868 US adults with and without T2D found diet quality (measured by the Healthy Eating Index score) to be inversely associated with physical activity levels^31^. Similarly, a 2009 study with 390 young adults (<20 years of age) with T2D found higher diet quality was to be associated with higher physical activity levels^32^. T2D behaviour modification interventions that focus on improving diet quality and physical activity in combination early after diagnosis need to be developed and tested.

Smoking is considered to be an important risk factor for arterial hypertension and diabetes management^33^. In the current study, those who did not improve their diet quality by 3 months were more likely to be current smokers than those who did improve their diet quality (*p* = 0.018). Similarly, a 2019 cross-sectional study of 229 people diagnosed with T2D in Brazil found that those with a lower diet quality (measured by the Healthy Eating Index score) were more likely to be current smokers than those with a higher diet quality^34^. These results suggest that individuals newly diagnosed with T2D who smoke may need extra support from healthcare professionals during patient education to improve diet quality.

This study found no demographic differences between those who improved their diet quality and those who did not. This may suggest demographic factors play no part in determining whether or not those newly diagnosed with T2D make early improvements to their diet quality, or there may be other factors that play a significantly larger role (e.g. BMI).

This study has some notable strengths and limitations. The biggest strength is that diet quality is tracked over time. A strength of the study sample is that the sample is broadly representative of the Australian population with T2D. The relatively low response rate obtained may have introduced non-response bias into the results, meaning they may not be indicative of the overall study population. However, it is important to note that the recruitment method was chosen for the ability to potentially reach a comprehensive national sample of people newly diagnosed with T2D. Email recruitment was used by Diabetes Australia for the first time in this study, thus the recruitment rate can only be estimated as it is unknown how many of the 14,108 people actually received and read the recruitment email sent. The figure of 2.9% as a response rate is therefore a minimum. Even though we sought to recruit as soon as people were diagnosed, there was a lag time between diagnosis and referral to Diabetes Australia and then the research team to interview, resulting in a mean of 114 days from diagnosis to baseline survey.

Unfortunately, people of Aboriginal and Torres Strait Islander descent were under-represented in our sample, as were people whose main language was not English. Dietary data were analysed using the DASH scoring system which is highly correlated with diabetes outcomes but does not describe detailed food patterns. People may have the same score but a different distribution of contributing components due to variance in food patterns^35^. Further, the use of a single 24-h dietary recall at each data collection point cannot account for day to day variations in participant’s dietary intake^36^. Multiple rounds of self-reported dietary intake data, physical activity data and physical measurements may have induced social desirability responses.

This paper explored initial changes in diet quality and the 3D study intends to investigate the outcomes associated with long-term improvements in diet quality. People newly diagnosed with T2D may require more support to improve their diet quality after diagnosis, especially current smokers. People in the 3D study who made early improvements to diet quality started with lower diet quality, higher rates of ex-smoking, higher physical activity levels and for males, a lower BMI. Future research should investigate how the diet quality changes people make around the time of diagnosis are related to long-term health outcomes.

## Supplementary information

Supplementary Figure 1

Supplementary Table 1

Supplementary Table 2
