# Supplementary material for: Short-term improvements in diet quality in people newly diagnosed with type 2 diabetes are associated with smoking status, physical activity and body mass index: the 3D case series study
Source: Nutr Diabetes. 2020 Jul 13;10:25. doi: 10.1038/s41387-020-0128-3 (PMC7359342; doi:10.1038/s41387-020-0128-3)
Supplement: Supplementary file 1 — Supplementary Figure 1 [file 41387_2020_128_MOESM1_ESM.docx]

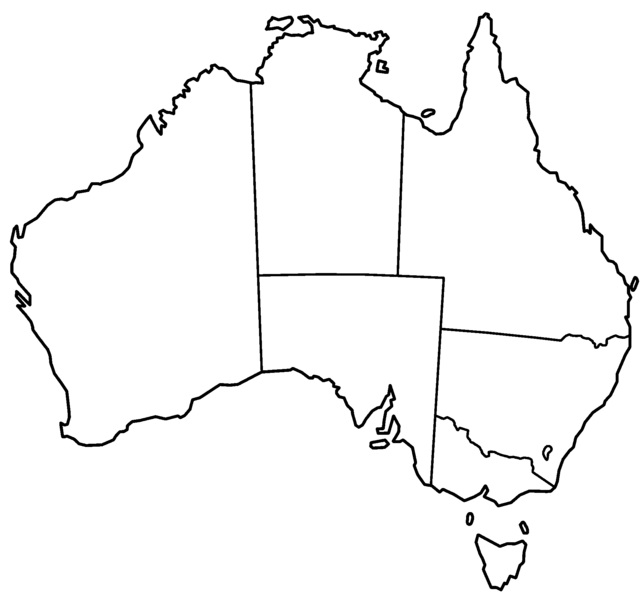


**NT**3D: 0.9%

Diabetes Aus: 1.2%

**QLD**

3D: 33.0%

Diabetes Aus: 19.1%

**WA**3D: 7.1%

Diabetes Aus: 10.0%

**SA**

3D: 8.0%

Diabetes Aus: 8.4%

**NSW**3D: 19.6%

Diabetes Aus: 32.3%

**ACT**3D: 2.7%

Diabetes Aus: 1.3%

**VIC**3D: 26.8%

Diabetes Aus: 25.6%

**TAS**3D: 1.8%

Diabetes Aus: 2.2%
