# Supplementary material for: Short-term improvements in diet quality in people newly diagnosed with type 2 diabetes are associated with smoking status, physical activity and body mass index: the 3D case series study
Source: Nutr Diabetes. 2020 Jul 13;10:25. doi: 10.1038/s41387-020-0128-3 (PMC7359342; doi:10.1038/s41387-020-0128-3)
Supplement: Supplementary file 2 — Supplementary Table 1 [file 41387_2020_128_MOESM2_ESM.docx]

| **Supplementary Table 1. Comparison of the baseline demographic characteristics of 3D study participants against other Australian cohorts** | | | | | |
| --- | --- | --- | --- | --- | --- |
| **Demographic characteristic** | **3D cohort** *n=*225  (100.0%) | **National cohort** *n=*1, 163, 403 (100.0%)^1^ | **LWD cohort** *n*=3, 951 (100.0%)^2^  n = 3, 951^2^ | **MILES-2 cohort** *n*=1, 264  (54.0%)^3^ | ***P* value** |
| **Sex n (%)**  Male  Female | 126 (56.0)  99 (44.0) | 636 179 (54.7)  527 224 (45.3) | 2175 (55.1) 1776 (44.9) | 725 (57.4) 539 (42.6) | 0.695^4^ 0.781^5^ 0.671^6^ |
| **Age n (%)**  <55 years  55-65 years  >65 years | 76 (33.7) 80 (35.6) 69 (30.7) | 281, 500 (24.2) 307, 815 (26.5) 574, 044 (49.3) | - | - | **<0.001^7^** |
| **Mean + SD age (years)** | 58.5 + 11.0 | - | 61.4 + 12.1 | 61 ± 9.0 | - |
| **Highest education** **n (%)**  Higher education degree  Diploma or certificate   No post-school education | 82 (37.0) 80 (36.0) 60 (27.0) | - | 516 (13.4) 1044 (27.2) 2284 (59.4) | 470 (37.3) 382 (30.3) 408 (32.4) | **<0.001^8^**  0.111^9^ |
| **Indigenous Australian n (%)**  Aboriginal or Torres Strait Islander   None | 2 (0.9) 223 (99.1) | - | 70 (1.8)  3878 (98.2) | 22 (1.7)  1242 (98.3) | 0.304^10^ 0.346^11^ |
| **Remoteness n (%)**  Major city  Inner regional   Outer regional  Remote/very remote | 172 (76.4)  31 (13.8) 17 (7.6)  5 (2.2) | 756, 310 (65.0) 251, 283 (21.6) 155, 810 (13.4) - | - | - | **<0.001^12^** |
| **Socioeconomic group n (%)**  1 (lowest SES)  2  3  4  5 | 45 (20.2) 48 (21.6) 42 (18.8) 44 (19.7) 44 (19.7) | 355, 218 (30.6)  219, 289 (18.8)  251, 351 (21.6)  193, 155 (16.6)  144, 391 (12.4) | - | - | **<0.001^13^** |
| **Living situation n (%)**  Partner/spouse  Other  Nobody | 148 (66.7)  30 (13.5)  44 (19.8) | - | - | - | - |
| **Gross yearly income n (%)**  <$50 000  $50 001 - $100 000  >$100 001 | 69 (36.1) 58 (30.4) 64 (33.5) | - | - | - | - |
| **Social class n (%)**  Upper/Middle  Working | 139 (79.0)  37 (21.0) | - | - | - | - |
| **Language n (%)** English speaking background  Main language at home | 212 (94.2)  210 (93.3) | - | - | - | - |
| SES = Socioeconomic status; LWD = Living with Diabetes; MILES-2 = Management and Impact for Long-term Empowerment and Success; - = data not available; ^1^ = data includes all Australians, both with and without diabetes; ^2^ = data includes Australian adults aged 18 years and over that had both type 1 (4.8%) and type 2 diabetes (95.2%); ^3^ = data includes Australians with type 2 diabetes only; ^4^ = Sex distribution representative with National cohort; ^5^ = Sex distribution representative with LWD cohort; ^6^ = Sex distribution representative with MILES-2 cohort; ^7^ = Age distribution not representative with National cohort; ^8^ = Highest education level not representative with LWD cohort; ^9^ = Highest education level representative with MILES-2 cohort; ^10^ =ATSI status representative with LWD cohort; ^11^ = ATSI status representative with MILES-2 cohort; ^12^ = Remoteness not representative with National cohort; ^13^ = Socioeconomic group not representative with National cohort.  Statistics: Chi-squared test for categorical variables. | | | | | |
