# Supplementary material for: Short-term improvements in diet quality in people newly diagnosed with type 2 diabetes are associated with smoking status, physical activity and body mass index: the 3D case series study
Source: Nutr Diabetes. 2020 Jul 13;10:25. doi: 10.1038/s41387-020-0128-3 (PMC7359342; doi:10.1038/s41387-020-0128-3)
Supplement: Supplementary file 3 — Supplementary Table 2 [file 41387_2020_128_MOESM3_ESM.docx]

| **Supplementary Table 2. Comparison of the baseline health characteristics of 3D study participants against other Australian cohorts** | | | | |  |
| --- | --- | --- | --- | --- | --- |
| **Health characteristic** | **3D cohort** *n=*225 (100.0%) | **LWD cohort** *n=*3, 951 (100.0%)^1^ | **MILES-2 cohort** *n*=1, 264 (54.0%)^2^ | ***P* value** | |
| **Mean + SD time since diagnosis (days)** | 114.5 + 41.1 | 13 140 + unknown | 4, 015 + unknown | - | |
| **Using diabetic medication n (%)** Yes   No | 145 (64.7)  79 (35.3) | - | 1088 (86.1)  176 (13.9) | **<0.001^3^** | |
| **BMI class n (%)**  Healthy  Overweight   Obese | 29 (13.1)  61 (27.5)  132 (59.4) | (16.9) (33.3) (49.7) | - (32.0) (53.0) | **0.017^4^** 0.179^5^ | |
| **Mean + SD BMI (kg/m^2^)**  Males  Females   Males and females | 32.6 + 6.9 30.9 + 7.4 31.7 + 7.2 | - - 31.1 + 6.9 | - - 32.8 + 6.4 | - | |
| **Waist circumference n (%)**  Meets recommendations  Exceeds recommendations | 20 (9.9) 183 (90.1) | - | - | - | |
| **Mean + SD waist circumference (cm)**  Males   Females   Males and females | 98.8 + 36.9 97.9 + 35.2 98.4 + 36.1 | - | - | - | |
| **Mean + SD weight (kg)**  Males  Females   Males and females | 96.1 + 19.2 87.0 + 19.3 92.8 + 19.8 | - | - | - | |
| **Mean + SD height (m)**  Males  Females   Males and females | 1.7 + 0.1 1.6 + 0.1 1.7 + 0.1 | - | - | - | |
| **Smoking status n (%)**  Current smoker   Ex-smoker   Never smoked | 22 (9.8)  65 (28.9) 138 (61.3) | - | - | - | |
| **Diagnosed with pre-diabetes n (%)**  Yes  No | 100 (45.5) 120 (54.5) | - | - | - | |
| **IPAQ n (%)** Low  Moderate  High | 78 (34.7) 122 (54.2)  25 (11.1) | - | - | - | |
| **K10 score n (%)**  Low  Moderate  High  Very high | 115 (51.6)  55 (24.7)  30 (13.4)  23 (10.3) | - | - | - | |
| LWD = Living with Diabetes; MILES-2 = Management and Impact for Long-term Empowerment and Success; IPAQ = International Physical Activity Questionnaire; cm = centimeters; kg = kilograms; BMI = Body Mass Index; m = meters; K10 = Kessler psychological distress scale; - = data not available; ^1^ = data includes all Australians, both with and without diabetes; ^2^ = data includes Australian adults aged 18 years and over that had both type 1 (4.8%) and type 2 diabetes (95.2%); ^3^ = Diabetic medication use not representative with MILES-2 cohort; ^4^ = BMI class not representative with LWD cohort; ^5^ = BMI class representative with MILES-2 cohort.  Statistics: Chi-squared test for categorical variables. | | | | | |
